# Generalized Hermite-Hadamard type inequalities involving fractional integral operators

**DOI:** 10.1186/s13660-017-1444-6

**Published:** 2017-07-19

**Authors:** Erhan Set, Muhammed Aslam Noor, Muhammed Uzair Awan, Abdurrahman Gözpinar

**Affiliations:** 1 Department of Mathematics, Faculty of Science and Arts, Ordu University, Ordu, Turkey; 2 Department of Mathematics, King Saud University, Riyadh, Saudi Arabia; 3 Department of Mathematics, COMSATS Institute of Information Technology, Park Road, Islamabad, Pakistan; 4 Department of Mathematics, Government College University, Faisalabad, Pakistan

**Keywords:** 26A33, 26D10, 26D15, 33B20, Hermite-Hadamard inequality, convex function, Hölder inequality, fractional integral operator

## Abstract

In this article, a new general integral identity involving generalized fractional integral operators is established. With the help of this identity new Hermite-Hadamard type inequalities are obtained for functions whose absolute values of derivatives are convex. As a consequence, the main results of this paper generalize the existing Hermite-Hadamard type inequalities involving the Riemann-Liouville fractional integral.

## Introduction and preliminaries

During the last century the theory of convexity has emerged as an interesting and fascinating field of mathematics. It plays a pivotal role in optimization theory, functional analysis, control theory and economics etc.

A function $f:I\subseteq\mathbb{R}\rightarrow\mathbb{R}$ is said to be convex if the inequality

$$\begin{aligned} f \bigl( tx+ ( 1-t ) y \bigr) \leq tf ( x ) + ( 1-t ) f ( y ) \end{aligned}$$

holds for all $x,y\in I $ and $t\in [ 0,1 ] $.

The following inequality is a so-called classical Hermite-Hadamard type inequality for convex functions. Let $f:I=[a,b]\subseteq\mathbb{R} \rightarrow\mathbb{R}$ be a convex function and $a,b\in I$ with $a< b$, then

1.1 $$\begin{aligned} f \biggl( \frac{a+b}{2} \biggr) \leq\frac{1}{b-a} \int_{a}^{b}f ( x ) \,dx\leq\frac{f ( a ) +f ( b ) }{2}. \end{aligned}$$

This inequality is one of the most useful inequalities in mathematical analysis. For new proofs, noteworthy extension, generalizations and numerous applications on this inequality, see, *e.g.*, [1–3] where further references are given.

The relationship between theory of convexity and theory of inequalities has motivated many researchers to study these theories in depth. As a consequence of this fact several inequalities have been obtained via convex functions; see [1].

The history of fractional calculus can be traced back to the letter of L’Hospital to Leibniz in which he inquired him about the notation he was using for the *n*th derivative of the linear function $f(x)=x$, $\frac{\mathrm{D}^{n}x}{\mathrm{D}x^{n}}$. L’Hospital asked the question: what would the result be if $n=\frac{1}{2}$. Leibniz replied: An apparent paradox, from which one day useful consequences will be drawn. Nowadays fractional calculus has become a powerful tool in many branches of mathematics. Sarikaya *et al.* [4] used the definitions of Riemannn-Liouville integrals and developed a new generalization of Hermite-Hadamard inequality. This result inspired many researchers to study this area. For more details, and for recent results and recently found properties concerning this operator one can consult [4–12].

We need some definition and mathematical preliminaries of fractional calculus theory for using in this study as follows.

### Definition 1.1

Let $f\in L[a,b]$. The Riemann-Liouville integrals $J_{a+}^{\alpha}f$ and $J_{b-}^{\alpha}f$ of order $\alpha> 0$ with $a\geq0$ are defined by

$$ J_{a+}^{\alpha}f(x)=\frac{1}{\Gamma(\alpha)} \int_{a}^{x}(x-t)^{ \alpha-1}f(t)\,dt,\quad x>a, $$

and

$$ J_{b-}^{\alpha}f(x)=\frac{1}{\Gamma(\alpha)} \int_{x}^{b}(t-x)^{ \alpha-1}f(t)\,dt,\quad x< b, $$

respectively. Here $\Gamma(t)$ is the Gamma function and its definition is $\Gamma(t)=\int_{0}^{\infty}e^{-x}x^{t-1}\,dx$. It is to be noted that $J_{a+}^{0}f(x)=J_{b-}^{0}f(x)=f(x)$; in the case of $\alpha=1$, the fractional integral reduces to the classical integral.

In [13], Zhu *et al.* established a new identity for differentiable convex mappings via the Riemann-Liouville fractional integral.

### Lemma 1.1

[13]

*Let* $f:[a,b]\rightarrow\mathbb{R}$ *be a differentiable mapping on* $(a,b)$ *with* $a< b$. *If* $f^{\prime}\in L[a,b]$, *then the following equality for fractional integrals hold*:

1.2 $$\begin{aligned}& \frac{\Gamma(\alpha+1)}{2(b-a)^{\alpha}}\bigl[\bigl(J_{\alpha^{-}}^{a}f \bigr) (b)+ \bigl(J_{\alpha^{+}}^{b}f\bigr) (a)\bigr]-f \biggl( \frac{a+b}{2} \biggr) \\& \quad =\frac{b-a}{2} \biggl[ \int_{0}^{1}k(t)f^{\prime}\bigl(ta+(1-t)b \bigr)\,dt- \int _{0}^{1}\bigl[(1-t)^{ \alpha}-t^{\alpha} \bigr]f^{\prime}\bigl(ta+(1-t)b\bigr)\,dt\biggr], \end{aligned}$$

*where*

$$k(t)= \textstyle\begin{cases} 1, & 0< t\leq\frac{1}{2}, \\ -1, & \frac{1}{2}< t\leq1. \end{cases} $$

Using the above identity, they gave the following result for the Riemann-Liouville fractional integral.

### Theorem 1.1

[13]

*Let* $f:[a,b]\rightarrow\mathbb{R}$ *be a differentiable mapping on* $(a,b)$ *with* $a< b$. *If* $\vert f^{\prime} \vert $ *is convex on* $[a,b]$, *then the following fractional inequality for fractional integrals holds*:

1.3 $$\begin{aligned}& \biggl\vert \frac{\Gamma(\alpha+1)}{ 2(b-a)^{\alpha}}\bigl[\bigl(J_{\alpha^{-}} ^{a}f\bigr) (b)+ \bigl(J_{\alpha^{+}}^{b}f\bigr) (a) \bigr]-f \biggl( \frac{a+b}{2} \biggr) \biggr\vert \\& \quad \leq\frac{b-a}{4(\alpha+1)} \biggl(\alpha+3-\frac{1}{2^{\alpha-1}} \biggr) \bigl[ \bigl\vert f^{\prime}(a) \bigr\vert + \bigl\vert f^{\prime }(b) \bigr\vert \bigr]. \end{aligned}$$

In [14], Raina introduced a class of functions defined formally by

1.4 $$ \mathcal{F}_{\rho,\lambda}^{\sigma}(x) = \mathcal{F}_{\rho,\lambda }^{\sigma(0),\sigma(1),\ldots} (x)= \sum_{k=0}^{\infty} \frac{ \sigma(k)}{\Gamma(\rho k+\lambda)}x^{k} \quad \bigl(\rho,\lambda>0; \vert x \vert < \mathbf{R}\bigr), $$

where the coefficients $\sigma(k)$ ($k\in\mathbb{N}=\mathbb{N} \cup\{0\}$) are a bounded sequence of positive real numbers and **R** is the set of real numbers. With the help of (1.4), Raina [14] and Agarwal *et al.* [15] defined the following left-sided and right-sided fractional integral operators, respectively:

1.5 $$\begin{aligned}& \bigl( \mathcal{J}_{\rho,\lambda,a+;w}^{\sigma}\varphi \bigr) (x)= \int_{a}^{x}(x-t)^{\lambda-1} \mathcal{F}_{\rho,\lambda}^{\sigma} \bigl[w(x-t)^{\rho}\bigr] \varphi(t)\,dt \quad (x>a>0), \end{aligned}$$

1.6 $$\begin{aligned}& \bigl( \mathcal{J}_{\rho,\lambda,b-;w}^{\sigma}\varphi \bigr) (x)= \int_{x}^{b}(t-x)^{\lambda-1} \mathcal{F}_{\rho,\lambda}^{\sigma} \bigl[w(t-x)^{\rho}\bigr] \varphi(t)\,dt \quad (0< x< b), \end{aligned}$$

where $\lambda,\rho>0$, $w\in\mathbb{R}$ and $\varphi(t)$ is such that the integral on the right side exists. Recently some new integral inequalities involving this operator have appeared in the literature (*see, e.g.*, [15–22]).

It is easy to verify that $\mathcal{J}_{\rho,\lambda,a+;w}^{\sigma }\varphi(x)$ and $\mathcal{J}_{\rho,\lambda,b-;w}^{\sigma}\varphi (x)$ are bounded integral operators on $L(a,b)$, if

1.7 $$ \mathfrak{M}:=\mathcal{F}_{\rho,\lambda+1}^{\sigma} \bigl[w(b-a)^{ \rho}\bigr] < \infty. $$

In fact, for $\varphi\in L(a,b)$, we have

1.8 $$ \bigl\Vert \mathcal{J}_{\rho,\lambda,a+;w}^{\sigma}\varphi(x) \bigr\Vert _{1} \leq \mathfrak{M} (b-a)^{\lambda} \Vert \varphi \Vert _{1} $$

and

1.9 $$ \bigl\Vert \mathcal{J}_{\rho,\lambda,b-;w}^{\sigma}\varphi(x) \bigr\Vert _{1} \leq \mathfrak{M} (b-a)^{\lambda} \Vert \varphi \Vert _{1}, $$

where

$$ \Vert \varphi \Vert _{p}:= \biggl( \int_{a}^{b} \bigl\vert \varphi(t) \bigr\vert ^{p} \,dt \biggr) ^{ \frac{1}{p}}. $$

Here, many useful fractional integral operators can be obtained by specializing the coefficient $\sigma(k)$. For instance the classical Riemann-Liouville fractional integrals $J_{a+}^{\alpha}$ and $J_{b-}^{\alpha}$ of order *α* follow easily by setting $\lambda=\alpha$, $\sigma(0)=1$ and $w=0$ in (1.5) and (1.6).

Motivated by the work in [13–15], firstly, we will prove a generalization of the identity given by Zhu *et al.* using generalized fractional integral operators. Then we will give some new Hermite-Hadamard type inequalities, which are generalizations of the results in [13] to the case $\lambda=\alpha$, $\sigma(0)=1$ and $w=0$. Our results can be viewed as a significant extension and generalization of the previously known results.

## Results and discussions

In this section, we derive our main results. For the sake of simplicity, we denote

$$ L_{f}(a,b;w;J):=\frac{1}{2(b-a)^{\lambda}} \bigl[ \bigl( \mathcal{J} ^{\sigma}_{\rho,\lambda,b^{-};w}f \bigr) (a)+ \bigl( \mathcal{J}^{ \sigma}_{\rho,\lambda,a^{+};w }f \bigr) (b) \bigr] -\mathcal{F}_{ \rho,\lambda+1}^{\sigma} \bigl[w(b-a)^{\rho}\bigr]f \biggl( \frac{a+b}{2} \biggr) . $$

### Lemma 2.1

*Let* $f :[a,b]\to\mathbb{R}$ *be a differentiable mapping on* $(a,b)$ *with* $a< b$. *If* $f^{\prime} \in L[a,b] $, *then the following equality for generalized fractional integral operators holds*:

$$\begin{aligned} L_{f}(a,b;w;J) =&\frac{(b-a)}{2} \biggl\{ \int_{0}^{1}k(t)f^{\prime}\bigl(ta+(1-t)b \bigr)\,dt \\ & {} - \int_{0}^{1}(1-t)^{\lambda} \mathcal{F}_{\rho,\lambda+1}^{\sigma} \bigl[w(b-a)^{\rho}(1-t)^{\rho} \bigr] f^{\prime} \bigl( ta+(1-t)b \bigr) \,dt \\ &{}+ \int_{0}^{1}t^{\lambda}\mathcal{F}_{\rho,\lambda+1}^{\sigma} \bigl[w(b-a)^{ \rho}t^{\rho}\bigr] f^{\prime} \bigl( ta+(1-t)b \bigr) \,dt \biggr\} , \end{aligned}$$

*where*

$$k(t)= \textstyle\begin{cases} \mathcal{F}_{\rho,\lambda+1}^{\sigma} [w(b-a)^{\rho}],& 0< t\leq \frac{1}{2}, \\ -\mathcal{F}_{\rho,\lambda+1}^{\sigma} [w(b-a)^{\rho}],& \frac{1}{2}< t\leq1, \end{cases} $$

$\rho,\lambda>0$, $w\in\mathbb{R}$.

### Proof

It suffices to note that

2.1 $$\begin{aligned} I =& \int_{0}^{\frac{1}{2}}\mathcal{F}_{\rho,\lambda+1}^{\sigma} \bigl[w(b-a)^{ \rho}\bigr]f^{\prime}\bigl(ta+(1-t)b\bigr)\,dt \\ & {} - \int_{\frac{1}{2}}^{1}\mathcal{F}_{\rho,\lambda+1}^{\sigma} \bigl[w(b-a)^{ \rho}\bigr]f^{\prime}\bigl(ta+(1-t)b\bigr)\,dt \\ &{}- \int_{0}^{1}(1-t)^{\lambda} \mathcal{F}_{\rho,\lambda+1}^{\sigma } \bigl[w(b-a)^{\rho}(1-t)^{\rho} \bigr] f^{\prime} \bigl( ta+(1-t)b \bigr) \,dt \\ &{}+ \int_{0}^{1}t^{\lambda}\mathcal{F}_{\rho,\lambda+1}^{\sigma} \bigl[w(b-a)^{ \rho}t^{\rho}\bigr] f^{\prime} \bigl( ta+(1-t)b \bigr) \,dt \\ :=&I_{1}+I_{2}+I_{3}+I_{4}. \end{aligned}$$

Changing variables with $x=ta+(1-t)b$, we get

$$\begin{aligned}& \begin{aligned} I_{1} &= \int_{0}^{\frac{1}{2}}\mathcal{F}_{\rho,\lambda+1}^{\sigma } \bigl[w(b-a)^{\rho}\bigr]f^{\prime}\bigl(ta+(1-t)b\bigr)\,dt \\ &=\frac{\mathcal{F}_{\rho,\lambda+1}^{\sigma} [w(b-a)^{\rho}]}{b-a} \biggl[ f(b)-f \biggl( \frac{a+b}{2} \biggr) \biggr] , \end{aligned} \\& \begin{aligned} I_{2} &=- \int_{\frac{1}{2}}^{1}\mathcal{F}_{\rho,\lambda+1}^{\sigma } \bigl[w(b-a)^{\rho}\bigr]f^{\prime}\bigl(ta+(1-t)b\bigr)\,dt \\ &=\frac{\mathcal{F}_{\rho,\lambda+1}^{\sigma} [w(b-a)^{\rho}]}{b-a} \biggl[ f(a)-f \biggl( \frac{a+b}{2} \biggr) \biggr] . \end{aligned} \end{aligned}$$

Integrating by parts, we have

2.2 $$\begin{aligned} I_{3} =&- \int_{0}^{1}(1-t)^{\lambda} \mathcal{F}_{\rho,\lambda+1} ^{\sigma} \bigl[w(b-a)^{\rho}(1-t)^{\rho} \bigr] f^{\prime} \bigl( ta+(1-t)b \bigr) \,dt \\ =&\frac{1}{b-a}(1-t)^{\lambda}\mathcal{F}_{\rho,\lambda+1}^{ \sigma} \bigl[w(b-a)^{\rho}(1-t)^{\rho}\bigr] f \bigl( ta+(1-t)b \bigr) \big|_{0} ^{1} \\ &{}+\frac{1}{b-a} \int_{0}^{1}(1-t)^{\lambda-1} \mathcal{F}_{\rho, \lambda}^{\sigma} \bigl[w(b-a)^{\rho}(1-t)^{\rho} \bigr]f \bigl( ta+(1-t)b \bigr) \,dt \\ =&-\frac{1}{b-a}\mathcal{F}_{\rho,\lambda+1}^{\sigma} \bigl[w(b-a)^{ \rho}\bigr]f(b) \\ &{}+\frac{1}{b-a} \int_{a}^{b} \biggl( \frac{x-a}{b-a} \biggr) ^{\lambda-1} \mathcal{F}_{\rho,\lambda}^{\sigma} \biggl[w(b-a)^{\rho} \biggl( \frac {x-a}{b-a} \biggr) ^{\rho}\biggr]\frac{f(x)}{b-a}\,dx \\ =&-\frac{1}{b-a}\mathcal{F}_{\rho,\lambda+1}^{\sigma} \bigl[w(b-a)^{ \rho}\bigr]f(b) +\frac{1}{(b-a)^{\lambda+1}} \bigl( \mathcal{J}^{\sigma} _{\rho,\lambda,b^{-};w}f \bigr) (a). \end{aligned}$$

Analogously

2.3 $$\begin{aligned} I_{4} =& \int_{0}^{1}t^{\lambda}\mathcal{F}_{\rho,\lambda+1}^{ \sigma} \bigl[w(b-a)^{\rho}t^{\rho}\bigr] f^{\prime} \bigl( ta+(1-t)b \bigr) \,dt \\ =&-\frac{1}{b-a}t^{\lambda}\mathcal{F}_{\rho,\lambda+1}^{\sigma} \bigl[w(b-a)^{\rho}t^{\rho}\bigr] f \bigl( ta+(1-t)b \bigr) \big|_{0}^{1} \\ &{}+\frac{1}{b-a} \int_{0}^{1}t^{\lambda-1}\mathcal{F}_{\rho,\lambda }^{\sigma} \bigl[w(b-a)^{\rho}t^{\rho}\bigr]f \bigl( ta+(1-t)b \bigr) \,dt \\ =&-\frac{1}{b-a}\mathcal{F}_{\rho,\lambda+1}^{\sigma} \bigl[w(b-a)^{ \rho}\bigr]f(a) \\ &{}+\frac{1}{b-a} \int_{a}^{b} \biggl( \frac{b-x}{b-a} \biggr) ^{\lambda-1} \mathcal{F}_{\rho,\lambda}^{\sigma} \biggl[w(b-a)^{\rho} \biggl( \frac {b-x}{b-a} \biggr) ^{\rho}\biggr]\frac{f(x)}{b-a}\,dx \\ =&-\frac{1}{b-a}\mathcal{F}_{\rho,\lambda+1}^{\sigma} \bigl[w(b-a)^{ \rho}\bigr]f(a) +\frac{1}{(b-a)^{\lambda+1}} \bigl( \mathcal{J}^{\sigma} _{\rho,\lambda,a^{+};w}f \bigr) (b). \end{aligned}$$

 □

Substituting the resulting equalities into equality (2.1), we have

2.4 $$\begin{aligned} I =&\frac{-2}{b-a}\mathcal{F}_{\rho,\lambda+1}^{\sigma} \bigl[w(b-a)^{ \rho}\bigr]f\biggl(\frac{a+b}{2}\biggr) \\ &{} +\frac{1}{(b-a)^{\lambda+1}} \bigl[ \bigl( \mathcal{J}^{\sigma}_{ \rho,\lambda,b^{-};w}f \bigr) (a)+ \bigl( \mathcal{J}^{\sigma}_{ \rho,\lambda,a^{+};w}f \bigr) (b) \bigr] . \end{aligned}$$

Thus, multiplying both sides by $\frac{(b-a)}{2}$, the result is obtained.

### Remark 2.1

Choosing $\lambda=\alpha$, $\sigma(0)=1$ and $w=0$ in Lemma 2.1, equality (2.1) reduces to equality (1.2).

### Theorem 2.1

*Let* $f :[a,b]\to\mathbb{R}$ *be a differentiable function on* $[a,b]$ *with* $a< b$. *If* $\vert f^{\prime} \vert $ *is convex on* $(a,b)$, *then the following inequality for generalized fractional integral operators holds*:

2.5 $$\begin{aligned} \bigl\vert L_{f}(a,b;w;J) \bigr\vert \leq& \frac{b-a}{4}\mathcal{F}_{\rho, \lambda+2}^{\sigma_{1}} \bigl[ \vert w \vert (b-a)^{\rho}\bigr] \bigl[ \bigl\vert f^{\prime }(a) \bigr\vert + \bigl\vert f^{\prime}(b) \bigr\vert \bigr] , \end{aligned}$$

*where*

$$ \sigma_{1}(k)=\sigma(k)\biggl( \lambda+\rho k+3-\frac{1}{2^{ \lambda+\rho k-1}} \biggr) , $$

$\rho,\lambda>0$, $w\in\mathbb{R}$, $s\in(0,1]$.

### Proof

Using Lemma 2.1 and convexity of $\vert f^{\prime} \vert $, we have

2.6 $$\begin{aligned}& \bigl\vert L_{f}(a,b;w;J) \bigr\vert \\& \quad \leq \frac{b-a}{2} \biggl\{ \biggl\vert \int_{0}^{\frac{1}{2}}\mathcal{F} _{\rho,\lambda+1}^{\sigma} \bigl[w(b-a)^{\rho}\bigr]f^{\prime}\bigl(ta+(1-t)b\bigr)\,dt \\& \quad\quad{} + \int_{\frac{1}{2}}^{1}\mathcal{F}_{\rho,\lambda+1}^{\sigma } \bigl[w(b-a)^{\rho}\bigr]f^{\prime}\bigl(ta+(1-t)b\bigr)\,dt \biggr\vert \\& \quad\quad{} + \biggl\vert \int_{0}^{1}(1-t)^{\lambda} \mathcal{F}_{\rho,\lambda+1} ^{\sigma} \bigl[w(b-a)^{\rho}(1-t)^{\rho} \bigr] f^{\prime} \bigl( ta+(1-t)b \bigr) \,dt \\& \quad\quad{} - \int_{0}^{1}t^{\lambda}\mathcal{F}_{\rho,\lambda+1}^{\sigma} \bigl[w(b-a)^{ \rho}t^{\rho}\bigr] f^{\prime} \bigl( ta+(1-t)b \bigr) \,dt \biggr\vert \biggr\} \\& \quad \leq \frac{b-a}{2} \Biggl\{ \int_{0}^{1} \bigl\vert \mathcal{F}_{\rho, \lambda+1}^{\sigma} \bigl[w(b-a)^{\rho}\bigr] \bigr\vert \bigl( t \bigl\vert f^{\prime}(a) \bigr\vert +(1-t) \bigl\vert f ^{\prime}(b) \bigr\vert \bigr) \,dt \\& \quad\quad{} +\sum_{k=0}^{\infty}\frac{\sigma(k) \vert w \vert ^{k}(b-a)^{\rho k}}{\Gamma (\rho k+\lambda+1)} \biggl[ \int_{0}^{\frac{1}{2}} \bigl((1-t)^{\lambda +\rho k}-t^{\lambda+\rho k} \bigr) \bigl( t \bigl\vert f^{\prime}(a) \bigr\vert +(1-t) \bigl\vert f^{\prime}(b) \bigr\vert \bigr) \,dt \\& \quad\quad{} + \int_{\frac{1}{2}}^{1} \bigl(t^{\lambda+\rho k}-(1-t)^{\lambda+ \rho k} \bigr) \bigl( t \bigl\vert f^{\prime}(a) \bigr\vert +(1-t) \bigl\vert f^{\prime}(b) \bigr\vert \bigr) \,dt \biggr] \Biggr\} \\& \quad \leq \frac{b-a}{2} \sum_{k=0}^{\infty} \frac{\sigma(k) \vert w \vert ^{k}(b-a)^{ \rho k}}{\Gamma(\rho k+\lambda+1)} \times \biggl\{ \biggl( \frac{ \vert f ^{\prime}(a) \vert + \vert f^{\prime}(b) \vert }{2} \biggr) \\& \quad\quad{} + \bigl\vert f^{\prime}(a) \bigr\vert \int_{0}^{\frac{1}{2}} \bigl(t(1-t)^{\lambda+\rho k}-t ^{\lambda+\rho k+1} \bigr)\,dt \\& \quad\quad{} + \bigl\vert f^{\prime}(b) \bigr\vert \int_{0}^{\frac{1}{2}} \bigl((1-t)^{\lambda+\rho k+1}-t ^{\lambda+\rho k}(1-t) \bigr)\,dt \\& \quad\quad{} + \bigl\vert f^{\prime}(a) \bigr\vert \int_{\frac{1}{2}}^{1} \bigl(t^{\lambda+\rho k+1}-t(1-t)^{ \lambda+\rho k} \bigr)\,dt \\& \quad\quad{} + \bigl\vert f^{\prime}(b) \bigr\vert \int_{\frac{1}{2}}^{1} \bigl((1-t)t^{\lambda+\rho k}-(1-t)^{ \lambda+\rho k+1} \bigr)\,dt \biggr\} \\& \quad = \frac{b-a}{2} \sum_{k=0}^{\infty} \frac{\sigma(k) \vert w \vert ^{k}(b-a)^{ \rho k}}{\Gamma(\rho k+\lambda+1)} \biggl[ \frac{1}{2}+\frac{1}{ \lambda+\rho k+1} \biggl( 1- \frac{1}{2^{\lambda+\rho k}} \biggr) \biggr] \bigl[ \bigl\vert f^{\prime}(a) \bigr\vert + \bigl\vert f^{\prime }(b) \bigr\vert \bigr] \\& \quad = \frac{b-a}{4}\sum_{k=0}^{\infty} \frac{\sigma (k) \vert w \vert ^{k}(b-a)^{ \rho k}}{\Gamma(\rho k+\lambda+2)} \biggl( \lambda+\rho k+3-\frac{1}{2^{ \lambda+\rho k-1}} \biggr) \bigl[ \bigl\vert f^{\prime}(a) \bigr\vert + \bigl\vert f^{\prime}(b) \bigr\vert \bigr] \\& \quad = \frac{b-a}{4}\mathcal{F}_{\rho,\lambda+2}^{\sigma_{1}} \bigl[ \vert w \vert (b-a)^{ \rho}\bigr] \bigl[ \bigl\vert f^{\prime}(a) \bigr\vert + \bigl\vert f^{\prime }(b) \bigr\vert \bigr] , \end{aligned}$$

using the facts that

$$\begin{aligned} \int_{0}^{\frac{1}{2}} \bigl(t(1-t)^{\lambda+\rho k}-t^{\lambda+ \rho k+1} \bigr)\,dt =& \int_{\frac{1}{2}}^{1} \bigl((1-t)t^{\lambda+ \rho k}-(1-t)^{\lambda+\rho k+1} \bigr)\,dt \\ =&\frac{2^{\lambda+\rho k+1}-(\lambda+\rho k+2)}{(\lambda+\rho k+1)( \lambda+\rho k+2)2^{\lambda+\rho k+1}} \end{aligned}$$

and

$$\begin{aligned} \int_{0}^{\frac{1}{2}} \bigl((1-t)^{\lambda+\rho k+1}-t^{\lambda+ \rho k}(1-t) \bigr)\,dt =& \int_{\frac{1}{2}}^{1} \bigl(t^{\lambda+ \rho k+1}-t(1-t)^{\lambda+\rho k} \bigr)\,dt \\ =& \frac{1}{\lambda+\rho k+2}-\frac{1}{(\lambda+\rho k+2)2^{\lambda +\rho k+2}} \\ &{}- \frac{\lambda+\rho k+3}{(\lambda+\rho k+1)(\lambda+\rho k+2) 2^{ \lambda+\rho k+2}}. \end{aligned}$$

Thus the proof is completed. □

### Remark 2.2

Choosing $\lambda=\alpha$, $\sigma(0)=1$ and $w=0$ in Theorem 2.1, inequality (2.5) reduces to inequality (1.3).

### Theorem 2.2

*Let* $f :[a,b]\to\mathbb{R}$ *be a differentiable function on* $(a,b)$ *with* $a< b$. *If* $\vert f^{\prime} \vert ^{q}$ *is convex and* $q>1$ *with* $\frac{1}{p}+\frac{1}{q}=1$, *then the following inequality for generalized fractional integral operators holds*:

2.7 $$\begin{aligned}& \bigl\vert L_{f}(a,b;w;J) \bigr\vert \\& \quad \leq \frac{b-a}{2} \biggl[\mathcal{F}_{\rho,\lambda+1}^{\sigma} \bigl[ \vert w \vert (b-a)^{ \rho}\bigr] \biggl( \frac{ \vert f^{\prime}(a) \vert ^{q}+ \vert f^{\prime}(a) \vert ^{q}}{2} \biggr) ^{ \frac{1}{q}} \\& \quad\quad{} +\mathcal{F}_{\rho,\lambda+1}^{\sigma_{2}} \bigl[ \vert w \vert (b-a)^{\rho}\bigr] \biggl( \biggl[ \frac{1}{8} \bigl\vert f^{\prime}(a) \bigr\vert ^{q}+\frac {3}{8} \bigl\vert f^{\prime}(b) \bigr\vert ^{q} \biggr] ^{\frac{1}{q}} + \biggl[ \frac{3}{8} \bigl\vert f^{\prime}(a) \bigr\vert ^{q}+\frac {1}{8} \bigl\vert f^{\prime}(b) \bigr\vert ^{q} \biggr] ^{\frac{1}{q}} \biggr)\biggr] \\& \quad \leq \mathcal{F}_{\rho,\lambda+1}^{\sigma_{3}} \bigl[ \vert w \vert (b-a)^{\rho}\bigr] \bigl[ \bigl\vert f^{\prime}(a) \bigr\vert + \bigl\vert f^{\prime }(b) \bigr\vert \bigr] , \end{aligned}$$

*where*

$$\begin{aligned}& \sigma_{2}(k)=\sigma(k) \biggl[ \frac{1}{p(\lambda+\rho k)+1} \biggl( 1- \frac{1}{2^{p( \lambda+\rho k)}} \biggr) \biggr] ^{\frac{1}{p}}, \\& \sigma_{3}(k)=\sigma(k) \biggl( \frac{1}{2} \biggr) ^{\frac{1}{q}} \biggl(1+\biggl[ \frac{4}{p( \lambda+\rho k)+1} \biggl( 1-\frac{1}{2^{p(\lambda+\rho k)}} \biggr) \biggr] ^{\frac{1}{p}}\biggr), \end{aligned}$$

$\rho,\lambda>0$ *and* $w\in\mathbb{R}$.

### Proof

By using Lemma 2.1, we have

2.8 $$\begin{aligned}& \bigl\vert L_{f}(a,b;w;J) \bigr\vert \\ & \quad \leq \frac{b-a}{2} \biggl\{ \biggl\vert \int_{0}^{\frac{1}{2}}\mathcal{F} _{\rho,\lambda+1}^{\sigma} \bigl[w(b-a)^{\rho}\bigr]f^{\prime}\bigl(ta+(1-t)b\bigr)\,dt \\ & \quad\quad{} + \int_{\frac{1}{2}}^{1}\mathcal{F}_{\rho,\lambda+1}^{\sigma} \bigl[w(b-a)^{ \rho}\bigr]f^{\prime}\bigl(ta+(1-t)b\bigr)\,dt \biggr\vert \\ & \quad\quad{} + \biggl\vert \int_{0}^{1}(1-t)^{\lambda} \mathcal{F}_{\rho,\lambda+1} ^{\sigma} \bigl[w(b-a)^{\rho}(1-t)^{\rho} \bigr] f^{\prime} \bigl( ta+(1-t)b \bigr) \,dt \\ & \quad\quad{} - \int_{0}^{1}t^{\lambda}\mathcal{F}_{\rho,\lambda+1}^{\sigma} \bigl[w(b-a)^{ \rho}t^{\rho}\bigr] f^{\prime} \bigl( ta+(1-t)b \bigr) \,dt \biggr\vert \biggr\} \\ & \quad \leq \frac{b-a}{2} \Biggl\{ \sum_{k=0}^{\infty} \frac{\sigma(k) \vert w \vert ^{k}(b-a)^{ \rho k}}{\Gamma(\rho k+\lambda+1)} \int_{0}^{1} \bigl\vert f^{\prime} \bigl(ta+(1-t)b\bigr) \bigr\vert \,dt \\ & \quad\quad{} +\sum_{k=0}^{\infty}\frac{\sigma(k) \vert w \vert ^{k}(b-a)^{\rho k}}{\Gamma (\rho k+\lambda+1)} \biggl[ \int_{0}^{\frac{1}{2}} \bigl((1-t)^{\lambda +\rho k}-t^{\lambda+\rho k} \bigr) \bigl\vert f^{\prime}\bigl(ta+(1-t)b\bigr) \bigr\vert \,dt \\ & \quad\quad{} + \int_{\frac{1}{2}}^{1} \bigl(t^{\lambda+\rho k}-(1-t)^{\lambda+ \rho k} \bigr) \bigl\vert f^{\prime}\bigl(ta+(1-t)b\bigr) \bigr\vert \,dt \biggr] \Biggr\} . \end{aligned}$$

Using the well-known Hölder inequality and convexity of $\vert f^{\prime} \vert ^{q}$ we get

2.9 $$ \int_{0}^{1} \bigl\vert f^{\prime} \bigl(ta+(1-t)b\bigr) \bigr\vert \,dt \leq \biggl( \frac{ \vert f^{\prime}(a) \vert ^{q}+ \vert f ^{\prime}(a) \vert ^{q}}{2} \biggr) ^{\frac{1}{q}}. $$

Thus

2.10 $$\begin{aligned}& \int_{0}^{\frac{1}{2}} \bigl((1-t)^{\lambda+\rho k}-t^{\lambda+ \rho k} \bigr) \bigl\vert f^{\prime}\bigl(ta+(1-t)b\bigr) \bigr\vert \,dt \\ & \quad \leq \biggl[ \int_{0}^{\frac{1}{2}} \bigl((1-t)^{\lambda+\rho k}-t ^{\lambda+\rho k} \bigr)^{p}\,dt \biggr] ^{\frac{1}{p}} \biggl[ \int_{0}^{ \frac{1}{2}} \bigl\vert f^{\prime} \bigl(ta+(1-t)b\bigr) \bigr\vert ^{q}\,dt \biggr] ^{\frac{1}{q}} \\ & \quad \leq \biggl[ \int_{0}^{\frac{1}{2}} \bigl((1-t)^{p(\lambda+\rho k)}-t ^{p(\lambda+\rho k)} \bigr)\,dt \biggr] ^{\frac{1}{p}} \biggl[ \int_{0}^{ \frac{1}{2}} \bigl( t \bigl\vert f^{\prime}(a) \bigr\vert ^{q}+(1-t) \bigl\vert f^{\prime}(a) \bigr\vert ^{q} \bigr) \,dt \biggr] ^{\frac{1}{q}} \\ & \quad = \biggl[ \frac{1}{p(\lambda+\rho k)+1} \biggl( 1-\frac{1}{2^{p(\lambda +\rho k)}} \biggr) \biggr] ^{\frac{1}{p}} \biggl[ \frac{1}{8} \bigl\vert f^{\prime}(a) \bigr\vert ^{q}+ \frac{3}{8} \bigl\vert f^{\prime}(b) \bigr\vert ^{q} \biggr] ^{\frac{1}{q}} \end{aligned}$$

and

2.11 $$\begin{aligned}& \int_{\frac{1}{2}}^{1} \bigl(t^{\lambda+\rho k}-(1-t)^{\lambda+ \rho k} \bigr) \bigl\vert f^{\prime}\bigl(ta+(1-t)b\bigr) \bigr\vert \,dt \\ & \quad \leq \biggl[ \int_{\frac{1}{2}}^{1} \bigl(t^{\lambda+\rho k}-(1-t)^{ \lambda+\rho k} \bigr)^{p}\,dt \biggr] ^{\frac{1}{p}} \biggl[ \int_{0}^{ \frac{1}{2}} \bigl\vert f^{\prime} \bigl(ta+(1-t)b\bigr) \bigr\vert ^{q}\,dt \biggr] ^{\frac{1}{q}} \\ & \quad \leq \biggl[ \int_{\frac{1}{2}}^{1} \bigl(t^{p(\lambda+\rho k)}-(1-t)^{p( \lambda+\rho k)} \bigr)\,dt \biggr] ^{\frac{1}{p}} \biggl[ \int_{ \frac{1}{2}}^{1} \bigl( t \bigl\vert f^{\prime}(a) \bigr\vert ^{q}+(1-t) \bigl\vert f^{\prime}(a) \bigr\vert ^{q} \bigr) \,dt \biggr] ^{\frac{1}{q}} \\ & \quad = \biggl[ \frac{1}{p(\lambda+\rho k)+1} \biggl( 1-\frac{1}{2^{p(\lambda +\rho k)}} \biggr) \biggr] ^{\frac{1}{p}} \biggl[ \frac{3}{8} \bigl\vert f^{\prime}(a) \bigr\vert ^{q}+ \frac{1}{8} \bigl\vert f^{\prime}(b) \bigr\vert ^{q} \biggr] ^{\frac{1}{q}}, \end{aligned}$$

where we used that $(A-B)^{p}\leq A^{p}-B^{p}$ for any $A\geq B \geq0$ and $p \geq1$ in (2.10) and (2.11).

Let $a_{1}=3 \vert f^{\prime}(a) \vert ^{q}$, $b_{1}= \vert f^{\prime}(b) \vert ^{q}$, $a_{2}= \vert f^{\prime }(a) \vert ^{q}$, $b_{2}=3 \vert f^{\prime}(b) \vert ^{q} $. Here $0 < \frac{1}{q}< 1$ for $q>1$. We use the fact that

$$ \sum_{k=1}^{n}(a_{k}+b_{k})^{s} \leq\sum_{k=1}^{n}a_{k}^{s}+ \sum_{k=1} ^{n}b_{k}^{s}. $$

For $0\leq s<1$, $a_{1},a_{2},a_{3},\ldots,a_{n}\geq0$, $b_{1},b_{2},b_{3},\ldots,b _{n}\geq0$. Combining the inequalities (2.10) with (2.11) we obtain

2.12 $$\begin{aligned}& \int_{0}^{\frac{1}{2}} \bigl((1-t)^{\lambda+\rho k}-t^{\lambda+ \rho k} \bigr) \bigl\vert f^{\prime}\bigl(ta+(1-t)b\bigr) \bigr\vert \,dt \\& \quad\quad{} + \int_{\frac{1}{2}}^{1} \bigl(t^{\lambda+\rho k}-(1-t)^{\lambda+ \rho k} \bigr) \bigl\vert f^{\prime}\bigl(ta+(1-t)b\bigr) \bigr\vert \,dt \\& \quad \leq \biggl[ \frac{1}{p(\lambda+\rho k)+1} \biggl( 1-\frac{1}{2^{p( \lambda+\rho k)}} \biggr) \biggr] ^{\frac{1}{p}} \biggl(\frac{1}{8}\biggr)^{ \frac{1}{q}} \\& \quad\quad{}\times \bigl( \bigl[ 3 \bigl\vert f^{\prime}(a) \bigr\vert ^{q}+ \bigl\vert f^{\prime}(b) \bigr\vert ^{q} \bigr] ^{ \frac{1}{q}} + \bigl[ \bigl\vert f^{\prime}(a) \bigr\vert ^{q}+3 \bigl\vert f^{\prime }(b) \bigr\vert ^{q} \bigr] ^{ \frac{1}{q}} \bigr) \\& \quad \leq \biggl[ \frac{1}{p(\lambda+\rho k)+1} \biggl( 1-\frac{1}{2^{p( \lambda+\rho k)}} \biggr) \biggr] ^{\frac{1}{p}} \biggl(\frac{1}{8}\biggr)^{ \frac{1}{q}} \bigl(3^{\frac{1}{q}}+1 \bigr) \bigl[ \bigl\vert f^{\prime}(a) \bigr\vert + \bigl\vert f^{\prime }(b) \bigr\vert \bigr] \\& \quad \leq \biggl[ \frac{1}{p(\lambda+\rho k)+1} \biggl( 1-\frac{1}{2^{p( \lambda+\rho k)}} \biggr) \biggr] ^{\frac{1}{p}} \biggl(\frac{1}{8}\biggr)^{ \frac{1}{q}}4 \bigl[ \bigl\vert f^{\prime}(a) \bigr\vert + \bigl\vert f^{\prime }(b) \bigr\vert \bigr] \\& \quad = \biggl[ \frac{4}{p(\lambda+\rho k)+1} \biggl( 1-\frac{1}{2^{p(\lambda +\rho k)}} \biggr) \biggr] ^{\frac{1}{p}} \biggl(\frac{1}{2}\biggr)^{\frac{1}{q}} \bigl[ \bigl\vert f^{\prime}(a) \bigr\vert + \bigl\vert f^{\prime }(b) \bigr\vert \bigr] \end{aligned}$$

and

2.13 $$ \biggl( \frac{ \vert f^{\prime}(a) \vert ^{q}+ \vert f^{\prime}(a) \vert ^{q}}{2} \biggr) ^{\frac{1}{q}} \leq\biggl( \frac{1}{2}\biggr)^{\frac{1}{q}} \bigl[ \bigl\vert f^{\prime}(a) \bigr\vert + \bigl\vert f^{\prime }(b) \bigr\vert \bigr] . $$

Thus putting the inequalities (2.9), (2.12) and (2.13) in (2.8), the proof is completed. □

### Corollary 2.1

*Choosing* $\lambda=\alpha$, $\sigma(0)=1$ *and* $w=0$ *in Theorem* 2.2, *inequality* (2.7) *becomes the following inequality*:

2.14 $$\begin{aligned}& \biggl\vert \frac{\Gamma(\alpha+1)}{2(b-a)^{\alpha}} \bigl[\bigl(J_{\alpha^{-}} ^{a}f\bigr) (b)+ \bigl(J_{\alpha^{+}}^{b}f\bigr) (a) \bigr]-f \biggl( \frac{a+b}{2} \biggr) \biggr\vert \\& \quad \leq \frac{b-a}{2} \biggl\{ \biggl[ \frac{ \vert f^{\prime}(a) \vert ^{q}+ \vert f^{\prime}(a) \vert ^{q}}{2} \biggr] ^{\frac{1}{q}} \\& \quad\quad{} + \biggl[ \frac{1}{\alpha p+1} \biggl( 1-\frac{1}{2^{\alpha p}} \biggr) \biggr] ^{\frac{1}{p}} \biggl( \biggl[ \frac{1}{8} \bigl\vert f^{\prime}(a) \bigr\vert ^{q}+\frac {3}{8} \bigl\vert f ^{\prime}(b) \bigr\vert ^{q} \biggr] ^{\frac{1}{q}} + \biggl[ \frac{3}{8} \bigl\vert f^{\prime}(a) \bigr\vert ^{q}+ \frac{1}{8} \bigl\vert f^{\prime}(b) \bigr\vert ^{q} \biggr] ^{\frac{1}{q}} \biggr) \biggr\} \\& \quad \leq \frac{b-a}{2} \biggl(1+ \biggl[ \frac{4}{\alpha p+1} \biggl(1- \frac{1}{2^{ \alpha p}} \biggr) \biggr] ^{\frac{1}{p}} \biggr) \biggl( \frac{1}{2} \biggr) ^{\frac{1}{q}} \bigl[ \bigl\vert f^{\prime}(a) \bigr\vert + \bigl\vert f^{\prime}(b) \bigr\vert \bigr] . \end{aligned}$$

## Conclusion

In this paper, we have obtained a new fractional integral identity. Utilizing this new identity as an auxiliary result, we have obtained some new variants of Hermite-Hadamard type inequalities. The results derived in this paper become natural generalizations of classical results. It is expected that the interested reader may find useful applications of these results and consequently this paper may stimulate further research in this area.
